# Functional and Antigen-Specific Serum Antibody Levels as Correlates of Protection against Shigellosis in a Controlled Human Challenge Study

**DOI:** 10.1128/CVI.00412-16

**Published:** 2017-02-06

**Authors:** Avital A. Shimanovich, Amanda D. Buskirk, Shannon J. Heine, William C. Blackwelder, Rezwanul Wahid, Karen L. Kotloff, Marcela F. Pasetti

**Affiliations:** a Department of Microbiology and Immunology, University of Maryland School of Medicine, Baltimore, Maryland, USA; b Department of Pediatrics, University of Maryland School of Medicine, Baltimore, Maryland, USA; c Center for Vaccine Development, University of Maryland School of Medicine, Baltimore, Maryland, USA; d Institute for Global Health, University of Maryland School of Medicine, Baltimore, Maryland, USA; Food and Drug Administration

**Keywords:** functional antibodies, Shigella, correlates of protection

## Abstract

Shigella is an important cause of diarrheal disease in young children living in developing countries. No approved vaccines are available, and the development of vaccine candidates has been hindered by the lack of firm immunological correlates of protection, among other reasons. To address this gap in knowledge, we established quantitative assays to measure Shigella-specific serum bactericidal antibody (SBA) and opsonophagocytic killing antibody (OPKA) activities and investigated their potential association with protection against disease in humans. SBA, OPKA, and Ipa-, VirG (IscA)-, and Shigella flexneri 2a lipopolysaccharide-specific serum IgG titers were determined in adult volunteers who received Shigella vaccine candidate EcSf2a-2 and in unvaccinated controls, all of whom were challenged with virulent Shigella flexneri 2a. Prechallenge antibody titers were compared with disease severity after challenge. SBA and OPKA, as well as IpaB- and VirG-specific IgG, significantly correlated with reduced illness. SBA and OPKA assays were also used to evaluate the immunogenicity of leading live attenuated vaccine candidates Shigella CVD 1204 and CVD 1208S in humans. A single oral immunization with CVD 1204 or CVD 1208S resulted in SBA seroconversion rates of 71% and 47% and OPKA seroconversion rates of 57% and 35%, respectively. Higher functional antibody responses were induced by CVD 1204, which is consistent with its lower attenuation. This is the first demonstration of SBA, OPKA, and IpaB- and VirG-specific IgG levels as potential serological correlates of protection against shigellosis in humans. These results warrant further studies to establish their capacity to predict protective immunity and vaccine efficacy.

## INTRODUCTION

Shigella causes a severe diarrheal and dysenteric disease that affects primarily young children living in low-resource settings (1). An estimated 188 million cases of shigellosis occur each year globally (2), and while the number of deaths in regions of endemicity have declined in recent decades (3), the burden of disease remains and continues to impair the health and quality of life of millions of underprivileged children. Development of a safe and effective vaccine is a promising strategy for control of shigellosis; however, despite decades of research, no approved vaccine is available (4). An important obstacle to vaccine development has been our limited understanding of the immunological mechanisms and host immune responses necessary to prevent infection and the lack of firm correlates of protective immunity.

Shigella-specific antibodies, particularly IgG, are believed to play an important role in host defenses against shigellosis. Children and adults living in areas where Shigella is endemic and who are exposed to this organism develop circulating antibody-secreting cells (ASCs) and serum IgG specific for the Shigella lipopolysaccharide (LPS) and invasion plasmid antigens (Ipas) (5–7). Seminal studies in Israeli soldiers identified an association between a reduced incidence of shigellosis and preexisting serum IgG specific for the LPS of the infecting serotype (6, 8, 9). During Shigella outbreaks, higher levels of LPS-specific serum IgG were found in asymptomatic individuals than in those who exhibited symptoms of disease (9). Systemic IgG responses induced by candidate vaccines have also been associated with protection against Shigella infection in animal models (10, 11). Further, B-cell knockout (KO) mice vaccinated with an attenuated Shigella strain succumbed to a lethal pulmonary challenge, whereas IgA KO mice resisted infection, indicating a requirement for antibodies other than IgA for protection (12, 13). Nonetheless, the mechanism by which antibodies, and IgG in particular, recognize and interact with Shigella *in vivo* to facilitate bacterial clearance and prevent disease remains unknown.

Based on the precedent of vaccine-induced functional antibodies representing serological correlates of protection against other bacterial pathogens (14), we hypothesized that antimicrobial activities of Shigella-specific antibodies, including complement-mediated killing and opsonophagocytosis, might be associated with clinical protection against shigellosis in humans. During convalescence, as well as after vaccination and long-term exposure, Shigella-specific antibodies (especially IgG) might mediate these antimicrobial functions through opsonization, followed by complement deposition and cell lysis, and/or by promoting phagocytosis and killing by innate immune cells via Fc receptor recognition. To investigate functional antibody responses and their potential associations with protection against shigellosis in humans, we established quantitative assays to measure Shigella-specific serum bactericidal antibody (SBA) and opsonophagocytic killing antibody (OPKA) activity and measured SBA and OPKA titers in sera from adult volunteers who had been orally immunized with a Shigella vaccine candidate (EcSf2a-2) or remained unvaccinated and were subsequently challenged with virulent Shigella flexneri 2a (15). In these same volunteers, we also measured levels in serum of IgG and IgG subclasses specific for S. flexneri LPS, IpaB, IpaC, IpaD, and VirG. Prechallenge antibody titers from each of these participants were compared with postchallenge disease outcomes to determine associations with clinical protection. To demonstrate the applicability of our functional assays to assess immune responses to vaccines, SBA and OPKA titers were measured in sera from human adult volunteers orally immunized with leading live attenuated vaccine candidates CVD 1204 (16) and CVD 1208S (17).

## RESULTS

### Optimization of quantitative SBA and OPKA assays.

**FIG 1:**
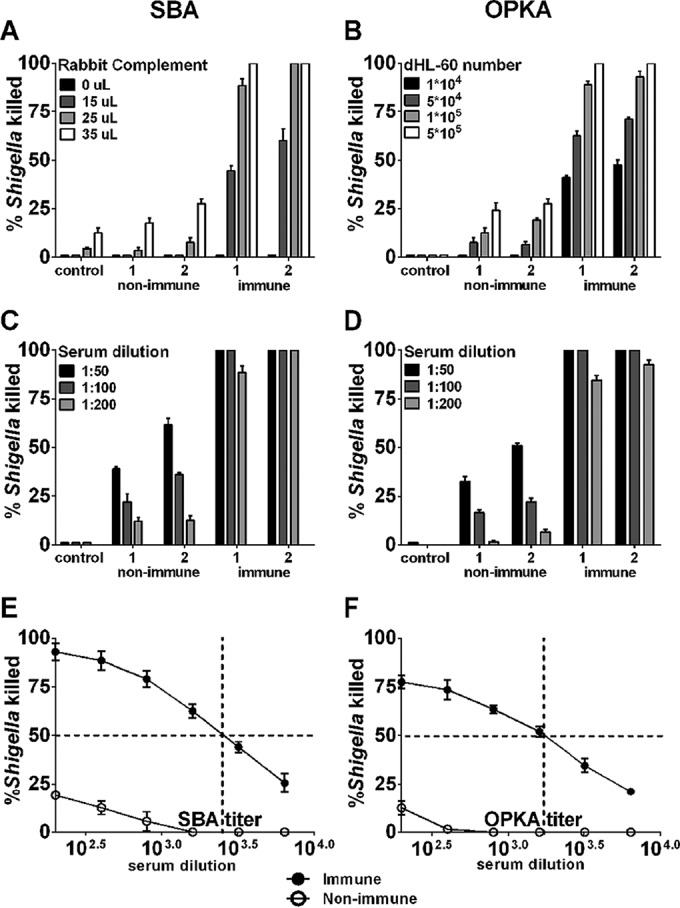
Shigella-specific serum bactericidal antibody (SBA) and opsonophagocytic killing antibody (OPKA) assay optimization. The percentages of organisms killed were determined using different amounts of baby rabbit complement (BRC) (A), DMF-differentiated HL60 cells (B), and serum dilutions (C and D) tested during assay optimization. Paired individual serum samples from two volunteers obtained before (nonimmune) and 28 days after (immune) immunization with Shigella vaccine strain CVD 1208S were used for assay optimization. Each assay included a negative control containing all reagents except serum. Curves show SBA (E) and OPKA (F) activity using the optimized assays; data represent mean percent killing ± SEM of four independent assays. The intersection of dashed lines exemplifies SBA and OPKA titers, calculated as the reciprocal of the serum dilution that produces 50% Shigella killing using Reed-Muench regression analysis.

We established SBA and OPKA assays to measure Shigella-specific functional antibodies in human sera. Paired serum samples from two CVD 1208S vaccine recipients obtained before (nonimmune) and after (immune) vaccination were used for assay optimization. To identify the best assay conditions, different amounts of complement (baby rabbit), bacteria (S. flexneri 2a 2457T), and HL-60 cells as well as multiple serum dilutions were tested (Fig. 1). Increasing amounts of complement (0 to 40%) in the SBA reaction resulted in higher bacterial killing; 25 μl (22%) allowed better discrimination of SBA activity between immune and nonimmune sera (Fig. 1A). Similarly, 1 × 10^5^ of dimethylformamide (DMF)-differentiated HL-60 cells allowed for a more sensitive determination of OPKA activity (Fig. 1 B). A starting serum dilution of 1:200 was selected for both assays to minimize nonspecific killing (Fig. 1C and D). Representative curves of SBA and OPKA activity for the positive and negative controls used throughout the study (in the final assay configuration) are shown in Fig. 1E and F. SBA and OPKA killing decreased proportionally with increasing serum dilutions. Almost negligible SBA and OPKA killing (≤20%) was observed in nonimmune (prevaccination) sera (Fig. 1E and F). The assays were reproducible, with coefficients of variations (CV) from eight independent experiments of 8.4% and 7.5% for SBA and OPKA, respectively (data not shown).

### Serum samples from a human Shigella challenge study used to investigate SBA and OPKA activity and correlation with clinical protection.

To determine a potential association between functional antibodies and protection against shigellosis, we examined sera from human adult volunteers who had received a hybrid Shigella-Escherichia coli EcSf2a-2 vaccine candidate (*n* = 15) and from nonvaccinated controls (*n* = 13), all of whom were experimentally challenged with S. flexneri 2a 2457T. SBA and OPKA titers were measured immediately before challenge and compared with clinical outcomes after challenge. In this clinical trial, the EcSf2a-2 vaccine provided only weak (and not statistically significant) protection against clinical illness: out of the 15 vaccine recipients, 7 were healthy or had mild disease, while the remaining 8 experienced moderate to severe disease (15). However, of the 13 unvaccinated controls, about half of them (*n* = 7) remained healthy or had mild disease after challenge. At the time the study was performed, this “natural” protection was attributed to preexisting immunity, as some of the volunteers were veterans who had likely been previously exposed to Shigella. Because this was an inpatient, controlled, human challenge study (one of few performed for Shigella in the last 20 years), volunteers were carefully observed and very detailed clinical data were available. Hence, despite the lack of vaccine efficacy *per se*, the study provided well characterized specimens and clinical information from volunteers who experienced a range of disease after experimental infection, making it suitable to explore associations between serological data and disease outcome.

### Higher prechallenge SBA and OPKA titers correlate with decreased disease severity.

**FIG 2:**
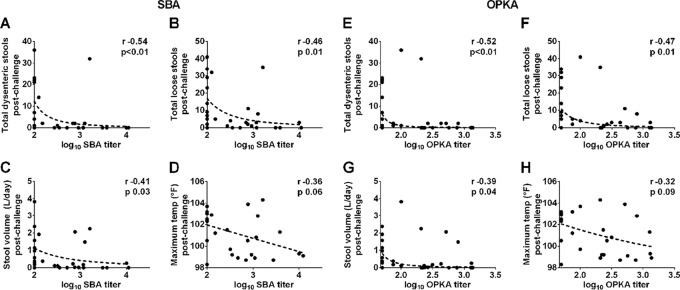
SBA and OPKA levels in serum correlate with reduced illness after Shigella infection. SBA and OPKA titers were measured in serum samples from U.S. adult volunteers immunized with Shigella vaccine EcSf2a-2 (*n* = 15) and nonvaccinated controls (*n* = 13) prior to challenge with virulent S. flexneri 2a. SBA and OPKA titers were compared with disease outcomes postchallenge: dysenteric stools (A and E), loose stools (B and F), stool volume (C and G), and temperature (D and H). Plots show Spearman's correlations (*r* value coefficient). For all analyses, a *P* value of ≤0.05 was considered significant.

**FIG 3:**
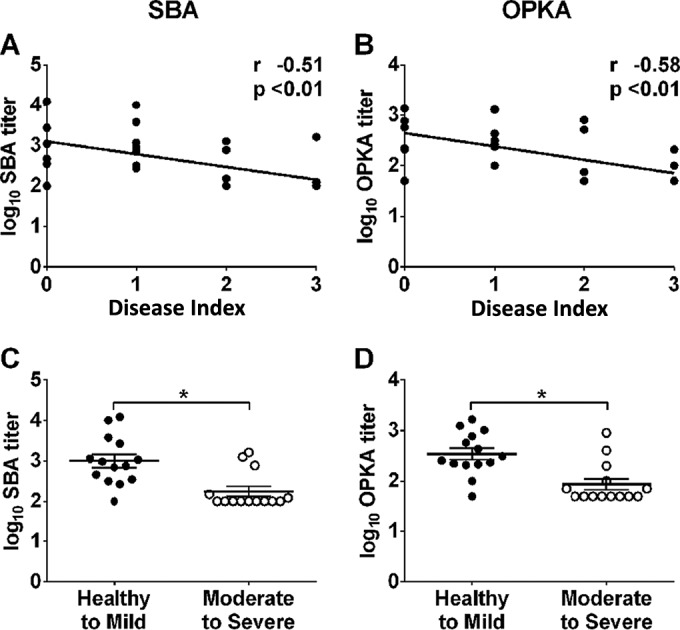
Elevated SBA and OPKA levels in serum are associated with decreased disease severity after Shigella infection. SBA (A) and OPKA (B) titers in EcSf2a-2 vaccine recipients (*n* = 15) and nonvaccinated controls (*n* = 13) were correlated with disease index (DI) postchallenge using Spearman's rank correlation (*r* and *P* values are indicated on the plots). The DIs after challenge were as follows: 6 of 28 volunteers remained healthy (DI of 0), 8 of 28 had mild disease (DI of 1), 8 of 28 experienced moderate disease (DI of 2), and 6 of 28 were severely ill (DI of 3). Titers were further grouped and compared based on disease severity (healthy to mild disease, DI of 0 or 1; moderate to severe disease, DI of 2 or 3) by using the Mann-Whitney U test (C and D). Asterisks indicate significant differences between groups at a *P* of ≤0.05.

SBA and OPKA titers measured in EcSf2a-2 vaccine recipients and unvaccinated controls at the time of challenge were examined for a potential association with four indicators of disease severity postchallenge: total numbers of dysenteric stools, loose stools, stool volume, and maximum body temperature (Fig. 2). Elevated SBA and OPKA titers were significantly associated with decreased numbers of dysenteric (Fig. 2A and E) and loose stools (Fig. 2B and F), as well as with lower stool volumes postchallenge (Fig. 2C and G). SBA and OPKA titers were also negatively but not significantly associated with maximum body temperatures postchallenge (Fig. 2D and H). SBA and OPKA titers were also compared among individuals who experienced different disease severities postchallenge based on disease index (DI) categories that take into account peak body temperature, daily number of bloody and loose stools, and total daily volume of the loose stools during the observation period (as described in Materials and Methods). This method provides a summary of disease outcome that is helpful to visualize associations with immune parameters. Both SBA and OPKA titers were significantly and negatively correlated with DI (Fig. 3A and B). Volunteers presenting as healthy or with mild disease after challenge (DI, 0 or 1) had higher SBA and OPKA titers at the time of challenge than those who experienced moderate or severe disease (DI, 2 or 3) (Fig. 3C and D). Importantly, results were in agreement regardless of whether DI or individual disease parameters were compared with antibody titers.

### Higher prechallenge IpaB- and VirG-specific IgG titers correlate with decreased disease severity.

**FIG 4:**
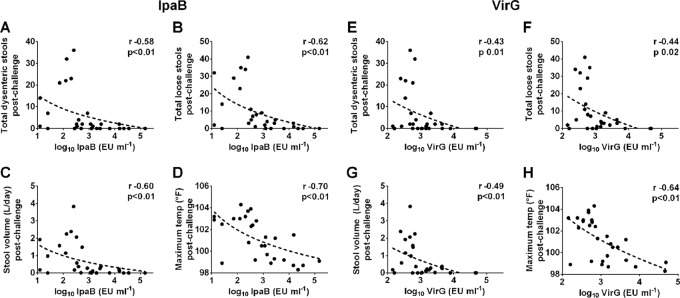
IpaB- and VirG-specific serum IgG titers correlate with reduced signs of shigellosis. IpaB- and VirG-specific IgG titers were measured in serum samples from both EcSf2a-2-vaccinated and nonvaccinated volunteers before challenge with wild-type S. flexneri 2a. IpaB- and VirG-specific IgG titers were compared with disease outcomes postchallenge: dysenteric stools (A and E), loose stools (B and F), stool volume (C and G), and temperature (D and H), using Spearman's rank correlation (*r* values indicated on the plots).

**FIG 5:**
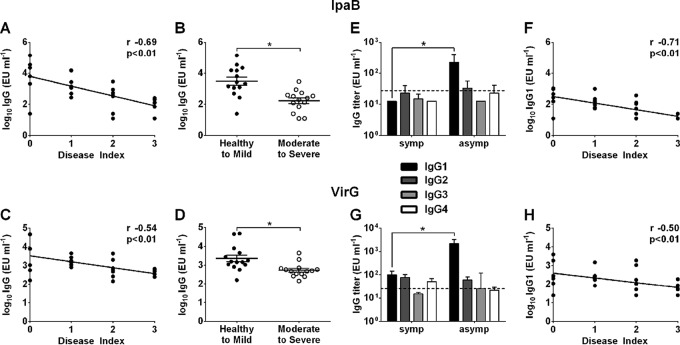
Antigen-specific IgG levels are associated with decreased disease severity postinfection. (A and C) IpaB (A)- and VirG (C)-specific serum IgG titers in EcSf2a-2 vaccine recipients and nonvaccinated controls were correlated with disease index (DI) postchallenge by using Spearman's rank correlation (*r* and *P* values indicated on the plots). Individuals included in each DI category are as described in the legend to Fig. 3. (B and D) Titers were further grouped and compared based on disease severity (healthy to mild disease, DI of 0 or 1; moderate to severe disease, DI of 2 or 3) by using the Mann-Whitney U test. Asterisks indicate significant differences between groups at a *P* of ≤0.05. (E and G) IpaB (E)- and VirG (G)-specific IgG1 to -4 subclasses were measured in serum samples from volunteers who experienced severe disease (DI, 3) or remained healthy (DI, 0) postchallenge; a dashed line indicates the limit of detection of the assay. (F and H) IpaB (F)- and VirG (H)-specific IgG1 titers were correlated with DI postchallenge using Spearman's rank correlation (*r* and *P* values indicated on the plots).

To determine whether antigen-specific antibodies were associated with protection, serum IgG titers against S. flexneri 2a LPS, IpaB, IpaC, IpaD, and VirG were measured in EcSf2a-2 recipients and in nonvaccinated controls before challenge, and results were compared with disease outcome. A significant negative correlation was observed between IpaB- and VirG-specific IgG levels and number of dysenteric (Fig. 4A and E) and loose stools (Fig. 4B and F), stool volume (Fig. 4C and G), and maximum body temperature (Fig. 4D and H). Elevated IpaB and VirG IgG levels were associated with decreased disease severity (Fig. 5A and C). Subjects with moderate to severe disease (DI, 2 or 3) had significantly lower IpaB- and VirG-specific IgG levels than those who remained healthy or experienced mild disease (DI, 0 or 1), as shown in Fig. 5B and D. We further examined the IpaB and VirG IgG subclass distribution and found that IpaB- and VirG-specific IgG1 titers were significantly increased in asymptomatic (DI, 0) subjects compared with those who were severely ill (DI, 3) (Fig. 5E and G), whereas IgG2, IgG3, and IgG4 were present at very low or negligible levels. IpaB- and VirG-specific IgG1 (but not IgG2 to -4) levels were correlated with decreased disease severity (Fig. 5F and H). IpaC and IpaD antibodies were detected prechallenge in 10 of the 28 enrolled subjects but levels were not correlated with disease outcome (data not shown). All serum samples were tested for S. flexneri 2a LPS-specific serum IgG, but no associations were observed between anti-LPS titers and any of the disease parameters postchallenge (see Fig. S1A to F in the supplemental material). A summary of all correlations between serum antibody titers and disease parameters (including *r* and *P* values) is given in Table S1 in the supplemental material.

### Correlations among antibody responses and their associations with disease severity.

**FIG 6:**
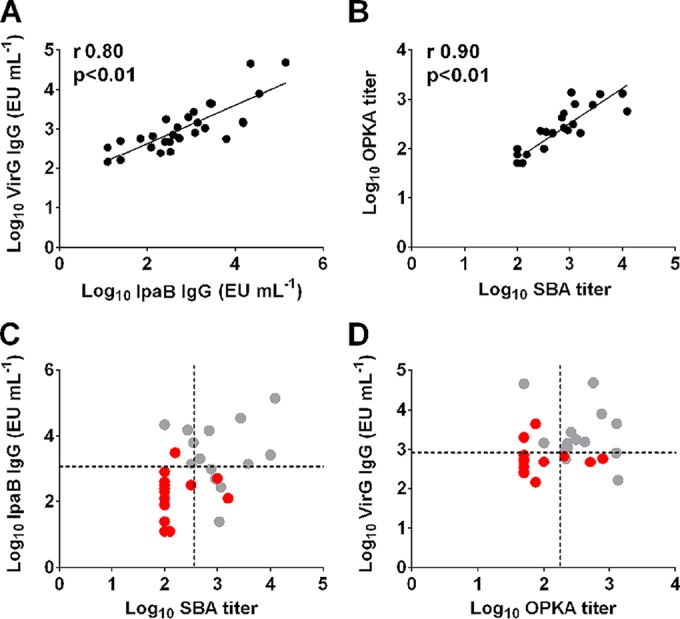
Serological trends associated with reduced disease severity. (A and B) Associations between IpaB- and VirG-specific serum IgG titers (A) and between SBA and OPKA titers (B) in EcSf2a-2 vaccine recipients and nonvaccinated controls prior to challenge. (C and D) Serological trends of IpaB and SBA titers (C) and VirG and OPKA titers (D) associated with disease severity. Gray circles represent individuals who remained healthy or had mild symptoms postchallenge (*n* = 14), and red circles represent those who experienced moderate to severe disease (*n* = 14). Dotted lines represent IpaB-specific IgG titers of ≥1,500 EU ml^−1^, VirG titers of ≥700 EU ml^−1^, and SBA and OPKA endpoint titers of ≥200.

VirG- and IpaB-specific IgG titers were significantly associated with each other (*r* = 0.80, *P* < 0.01) (Fig. 6A). Likewise, SBA and OPKA titers were significantly associated (*r* = 0.90, *P* < 0.01) (Fig. 6B). In a further examination of coassociations between antigen-specific IgG, functional antibody titers, and disease outcome, three serological trends were identified in volunteers who remained healthy or experienced mild disease after challenge (DI, 0 or 1): (i) elevated IpaB-specific IgG (≥1,500 ELISA units [EU] ml^−1^), (ii) elevated SBA titers (≥200), or (iii) both (Fig. 6C). The presence of both elevated IpaB and SBA titers was the most stringent, not met by any of the volunteers who experienced moderate to severe disease (Fig. 6C, upper right quadrant), the majority of whom had IpaB titers below 1,500 EU ml^−1^ and SBA endpoint titers below 200 (Fig. 6C, lower left quadrant). The same trends applied to OPKA and VirG: volunteers who remained healthy or experienced mild disease after challenge had elevated OPKA titers (≥200), elevated VirG-specific serum IgG titers (≥700 EU ml^−1^), or both (Fig. 6D), as well as elevated titers of the combinations SBA-VirG and OPKA-IpaB (data not shown).

### Multiple logistic regression modeling of disease severity.

Logistic regression models were fit to disease severity, collapsed to two categories (less severe for a DI of 0 or 1, more severe for a DI of 2 or 3). Previous EcSf2a-2 vaccinations were almost equally prevalent in these two groups (7 of 14 individuals with less severe disease and 8 of 14 with more severe disease), so vaccination status was not included in the modeling. As explained above, EcSf2a-2 provided only weak and not statistically significant protection against clinical illness after challenge (15). After consideration of log_10_-transformed prechallenge LPS, SBA, OPKA, IpaB, and VirG as covariates, higher IpaB (*P* = 0.03) and SBA (*P* = 0.03) titers remained associated with less severe disease. In another model, higher OPKA (*P* = 0.02) and VirG (*P* = 0.04) titers were significantly associated with less severe disease. These results are consistent with the observed trends described above (Fig. 6C and D).

### Functional and antigen-specific antibody responses postchallenge.

**FIG 7:**
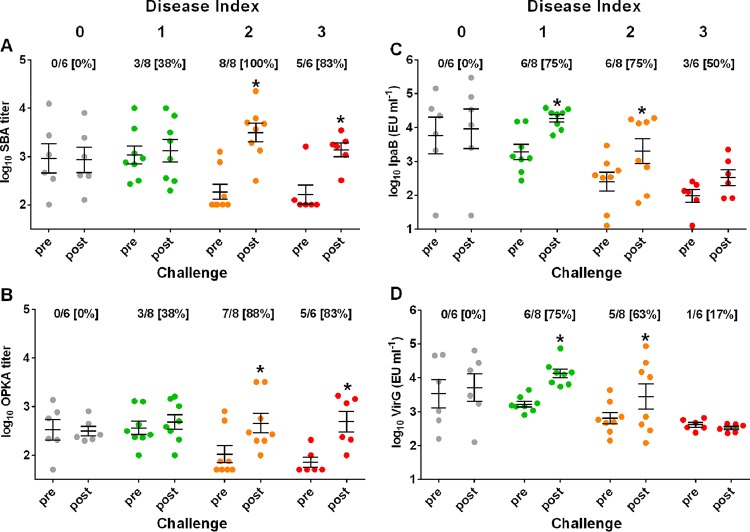
Functional and antigen-specific antibodies induced after S. flexneri 2a challenge. SBA (A), OPKA (B), and IpaB (C)- and VirG (D)-specific IgG titers before and 28 days after challenge in groups with different disease indexes. Data represent individual titers from all challenged individuals (*n* = 28), with the mean ± SEM indicated. Asterisks indicate statistically significant differences as determined by the Mann-Whitney U test (*P* ≤ 0.05). The numbers below the DIs at the top of each graph represent the number of subjects that seroconverted (≥2-fold SBA and OPKA titers and ≥4-fold IpaB- and VirG-specific IgG titers) over the total number of subjects, with the percent seroconversion shown in brackets.

SBA and OPKA titers (Fig. 7A and B), as well as IpaB- and VirG-specific IgG levels (Fig. 7C and D), were also measured in all subjects 28 days postchallenge to determine the breadth of serological responses following infection in relation to preexisting immunity and disease outcome. Postchallenge titers were compared with those measured immediately before challenge, and seroconversion rates were calculated for each DI group. We hypothesized that individuals who experienced severe illness (indicative of a stronger infection) would respond more vigorously than those who had no or mild disease. Indeed, SBA and OPKA titers increased significantly only in individuals who experienced moderate to severe disease (DI, 2 or 3), with seroconversion rates between 83 and 100% (Fig. 7A and B), whereas no seroconversions were detected among those who remained healthy (DI, 0). Healthy individuals also failed to respond to IpaB and VirG. However, different from the functional antibody responses, significant increases in IpaB and VirG titers after challenge were seen in subjects with mild disease (DI, 1), as well as in those with moderate disease (DI, 2), but not in those with severe disease (DI, 3), although half of the severely ill individuals seroconverted for IpaB IgG (Fig. 7C and D). In summary, preexisting protective immunity hindered serological responses postchallenge, and the strength of bacterial infection influenced the type of antibodies produced, favoring increases in antibodies with functional antimicrobial activity.

### SBA and OPKA after oral vaccination with live attenuated Shigella.

**FIG 8:**
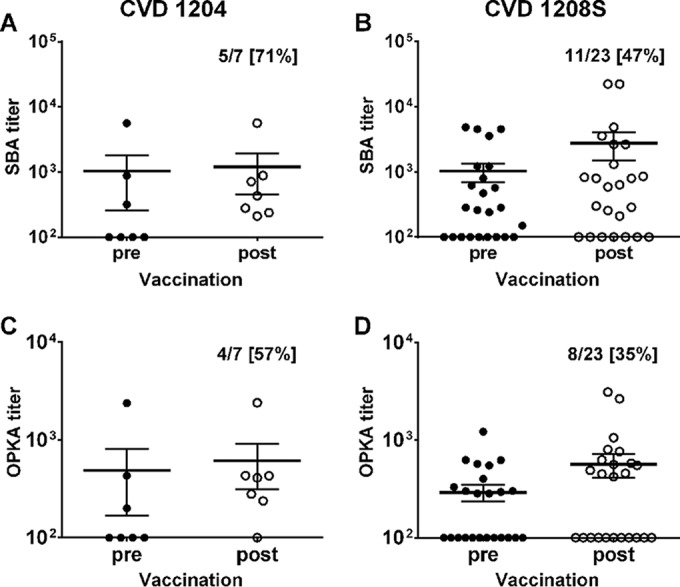
SBA and OPKA antibody responses induced by oral vaccination with live attenuated S. flexneri 2a vaccines. SBA (A and B) and OPKA (C and D) titers in serum samples from adult U.S. volunteers orally immunized with 10^7^ CFU of CVD 1204 (*n* = 7) or CVD 1208S (*n* = 23) vaccine strains before and 28 days after vaccination. The numbers in the upper right of each graph are the number of subjects that seroconverted over the total number of subjects, with the percent seroconversion shown in brackets.

To investigate the capacity of leading vaccine candidates to induce functional antibodies and to demonstrate the applicability of these functional assays to discern candidates based on their immunogenicity, SBA and OPKA titers were measured in serum samples from adult volunteers who received a single oral dose of live attenuated vaccine strains CVD 1204 (*n* = 7) or CVD 1208S (*n* = 23) in Center for Vaccine Development (CVD) clinical studies, before and 28 days after vaccination (16, 17). Both studies were performed in adult community volunteers, at the same clinical site (CVD, Baltimore, MD), and by the same research team. Prior to vaccination, about half of the enrolled (17 of 30) subjects had positive SBA titers (endpoint, ≥200) (Fig. 8). Among the CVD 1204 recipients, response rates 28 days postvaccination were 71% (5 of 7) for SBA and 57% (4 of 7) for OPKA (Fig. 8A and C); except for one subject (the same in both assays), vaccine-induced SBA and OPKA responses were seen in individuals who had SBA and OPKA titers below an endpoint titer of 200. Following CVD 1208S vaccination, the response rates were somewhat lower, i.e., 47% (11 of 23) for SBA and 35% (8 of 23) for OPKA (Fig. 8B and D), but were also observed mainly in subjects who had low prevaccination titers. Subjects who had SBA titers below 200 (endpoint) before or after vaccination also had OPKA titers of <200 (endpoint). SBA and OPKA were tested in placebo recipients included in the CVD 1208S study, and none had detectable responses (data not shown). These results confirm the suitability of SBA and OPKA to evaluate and even discriminate vaccines based on their capacity to elicit functional humoral immunity.

## DISCUSSION

In this study, we reported the successful establishment of assays to measure Shigella SBA and OPKA endpoint titers in human sera, the determination of SBA and OPKA responses in adult volunteers following vaccination and experimental infection, and a detailed analysis of SBA and OPKA antibody levels and clinical outcome in volunteers who participated in a controlled, nonrandomized, human challenge study. The immune effectors necessary to prevent Shigella infection and the mechanisms involved are not fully understood. We demonstrated, for the first time, a significant association between serological Shigella-specific functional antibody activity and protection against shigellosis. Bactericidal and macrophage phagocytic activities have been detected in sera from infected individuals living in regions of Shigella endemicity (18–21). However, their association with clinical protection had never been investigated. Antibody-dependent complement-mediated killing and opsonophagocytic activity have been associated with protection against other bacterial pathogens (22–24). Our results extend the value of functional antibody measurements as indicators of protective immunity against Shigella.

The SBA assay was more sensitive in detecting responses postchallenge and postvaccination. It also had more potential than the OPKA assay to predict the absence of severe disease after challenge, i.e., a higher rate of volunteers who remained healthy or experienced mild disease had elevated prechallenge SBA titers (93%) than OPKA titers (86%); any serum sample that was negative for SBA was also negative for OPKA, but the reverse was not true. One possible explanation for these observations is the greater stringency of the OPKA assay, which involves antibody-mediated opsonization, complement deposition, phagocytosis, and killing, as opposed to only complement activation and killing in the SBA assay.

Along with SBA and OPKA titers, antigen-specific IgG titers were measured in challenged volunteers. To our knowledge, we are the first to demonstrate a correlation between elevated anti-VirG and IpaB IgG1 levels and reduced illness following Shigella infection in humans. Immunization with IpaB has been shown to protect mice challenged with virulent Shigella bacteria via the pulmonary route (including challenge with heterologous serotypes), and IpaB, along with IpaD, constitutes the basis for a new Shigella subunit vaccine candidate (11, 25). Our results further support the hypothesis that IpaB-specific immunity is relevant for protection in humans. Conversely, we did not find any association between IpaD antibodies and clinical protection. However, only a small fraction of the subjects included in our study had detectable IpaD-specific IgG, and these levels were very low. Results from animal models suggest that the contribution of IpaD to protection might be through induction of robust gamma interferon (IFN-γ)-secreting T cells rather than antibodies (26). We did not anticipate finding a direct correlation between antibodies to VirG and reduced illness. Although regarded as an important virulence factor in Shigella pathogenesis, VirG is not considered a protective antigen. In fact, VirG has been deleted in several candidate live-vaccine strains (4). We have preliminary evidence that suggests that VirG elicits protective immunity in mice immunized intranasally and challenged via the pulmonary route with S. flexneri 2a (A. A. Shimanovich and M. F. Pasetti, unpublished data).

Interestingly, elevated IpaB- and VirG-specific IgG (not SBA and OPKA) titers are significantly correlated with reduced body temperature following experimental infection. Although serological associations do not imply causality, these antibodies might conceivably block cell invasion and bacterial spread, thus averting the acute release of proinflammatory cytokines (e.g., tumor necrosis factor alpha) that cause fever (27, 28). IgG1 was the main subclass directed against IpaB and VirG; this molecule is known to have a strong capacity for microbial opsonization, which is compatible with such a role. VirG- and IpaB-specific IgG antibodies were found in stools from Shigella-infected children enrolled in the GEMS study (A. Buskirk, presented at the 2016 Annual Conference of the U.S-Japan Panel on Cholera and Other Bacterial Enteric Infections, Bethesda, MD, 11 to 15 January 2016), which suggests their contribution to local defenses. How IgG reaches the gastrointestinal lumen (i.e., whether it is produced locally or it transudes from circulation) is not known. A case has been made for systemic LPS-specific IgG induced by parenteral immunization transuding across the epithelial lining for protection against Shigella via complement activation and bactericidal activity (29, 30). Whether the antibodies we measured in serum have an actual protective role or are merely surrogates of protection remains to be explored.

Subjects who remained healthy in our study had distinct serological profiles: elevated levels of SBA and OPKA (≥200 dilution titer), elevated levels of IpaB- and VirG-specific IgG (≥1,500 EU/ml), or both. Early studies linked the presence of LPS antibodies with serotype-specific resistance to shigellosis among Israeli soldiers (6, 8, 9). We did not find a significant association between S. flexneri 2a LPS-specific IgG titers and reduced disease in our study. This could be due to differences in the populations studied, ours being U.S. adult volunteers whose exposure to Shigella is rare as opposed to individuals living in areas of endemicity who develop high levels of anti-LPS antibodies as a result of repeated encounters with the organism (5, 6, 31). It has been acknowledged, however, that levels of circulating anti-LPS only partly reflect naturally acquired protective immunity and that rather the length of exposure more strongly associates with reduced incidence of disease due to additional immune components involved (32). This is in agreement with our findings of IpaB- and VirG-specific serum antibodies being associated with protection.

An important question that ensues is the specificity of antibodies that mediate the SBA and OPKA activities we measured. Lin et al. reported SBA activity of mouse monoclonal antibodies against Shigella O polysaccharide (OPS) (33), and SBA responses were induced by a Shigella-OPS conjugate vaccine in adult volunteers in a recent phase I study (40). Similarly, anti-LPS and -OPS antibodies are known to promote bactericidal and opsonophagocytic killing against other pathogens (34, 41, 42). We performed pilot experiments to address this question and observed that SBA killing and OPKA killing were reduced, although not completely, by depletion of anti-LPS antibodies, which suggests the involvement of other antibody specificities in these antimicrobial functions. Shigella outer membrane proteins (OMP) have been shown to elicit Shigella SBA activity in mice (35). The small amounts of serum available from our challenged volunteers precluded extensive antigen specificity analyses. Additional Shigella vaccine/challenge studies are forthcoming, which will enable us to further investigate the serological responses reported here.

An interesting observation was the striking increase in SBA and OPKA in subjects who experienced more severe disease postchallenge, while IpaB and VirG IgG increased mainly in subjects who had milder disease. Also notable is the absence of serological responses in subjects similarly exposed to the organisms but who remained healthy postchallenge. These results suggest a differential priming of functional versus antigen-specific antibodies on the basis of bacterial invasion and/or host-pathogen interaction that is worth exploring in future challenge studies. Such knowledge can inform the design of vaccines to induce relevant humoral immunity and their clinical evaluation.

Another novel contribution of our work is the demonstration of SBA and OPKA responses in volunteers orally immunized with Shigella vaccine strains CVD 1204 or CVD 1208S. The higher rate of SBA and OPKA responses among subjects that received CVD 1204, rather than CVD 1208S, likely reflects the higher reactogenicity of CVD 1204 and is in agreement with other immunologic readouts of this vaccine (17). Based on the associations described, it would be expected for these vaccines to induce measurable protective immunity. Thus, these functional assays can distinguish vaccine candidates in terms of their immunogenic (and potentially protective) capacity. Future human challenge studies will provide an opportunity to determine the suitability of these assays to predict vaccine efficacy and establish serological thresholds indicative of protection.

In conclusion, we have demonstrated that SBA and OPKA provide a measure of functional antibody activity associated with reduced shigellosis in humans and that, along with IpaB and VirG serum IgG, are potential immune correlates of protection. These results warrant further analysis to determine the usefulness of these serological measurements to predict protective immunity and vaccine efficacy.

## MATERIALS AND METHODS

### Clinical studies and serum samples.

The serum samples examined in this study were obtained through a series of clinical studies performed in healthy community volunteers at the Center for Vaccine Development (CVD). (i) We performed a phase IIB Shigella challenge study that included 15 adult subjects previously vaccinated with EcSf2a-2, an E. coli K-12 *aroD* mutant vaccine strain carrying the S. flexneri 5a virulence plasmid (encoding the type III secretion system and intracellular spread proteins) and chromosomal genes encoding the S. flexneri 2a O polysaccharide, as well as 13 newly recruited unvaccinated controls (15). The vaccinated group received four oral doses of EcSf2a-2 (7 × 10^8^ CFU per dose) on days 0, 3, 14, and 17 (15). One month after the last vaccine dose, these individuals as well as the unvaccinated controls (*n* = 28 total) were orally fed 1 × 10^3^ CFU of wild-type strain S. flexneri 2a 2457T; detailed challenge procedures are described elsewhere (15). Disease symptoms and signs, including body temperature, stool consistency, and presence of blood, were monitored during the 5-day inpatient observation period following challenge, after which all volunteers received a full course of ciprofloxacin. We used a categorical outcome-based postchallenge disease index (DI) based on peak oral body temperature, daily number of bloody and loose stools, and total daily volume of stools during the observation period, as previously described (36). Based on severity, bloody stools, loose stools, stool volume, and maximum body temperature, scores of 1 to 3 were assigned. A score of 0 was assigned to subjects who did not have bloody stools or loose stools or who had a body temperature of ≤100.2°F; a score of 1 was assigned to those with 1 to 2 bloody stools, 1 to 2 loose stools, a stool volume of ≤500 ml, or a temperature of ≤102.0°F; a score of 2 was assigned to those with 3 to 10 bloody stools, 3 to 10 loose stools, a stool volume of ≤2 liters, or a temperature between 102.0 and 104.0°F; and a score of 3 was assigned to those with >10 bloody stools, >10 loose stools, a stool volume of >2 liters, or a temperature of >104°F. The DI was defined based on the sum of these scores, which ranged from 0 to 12, for each individual. A DI of 0 was assigned to individuals with total scores of 0; a DI of 1 (mild disease) was assigned to subjects with total scores of 1 to 4; a DI of 2 (moderate disease) was assigned to those with total scores of 5 to 9; and a DI of 3 was assigned to subjects with total scores of 10 to 12. Serum samples were obtained before as well as 28 days after challenge. (ii) We performed a phase I clinical study in which volunteers received a single oral dose (1 × 10^7^ CFU) of candidate vaccine CVD 1204 (*n* = 7), an S. flexneri 2a Δ*guaBA* mutant strain which harbors deletions in the guanine nucleotide synthesis pathway, or placebo (*n* = 2) (16). (iii) We performed phase I and phase II studies in which volunteers received an oral dose of candidate vaccine CVD 1208S (*n* = 23), an S. flexneri 2a strain 2457T Δ*guaBA* Δ*sen* Δ*set* mutant which harbors additional attenuation conferred by deletions in the genes encoding Shigella enterotoxins (ShETs) 1 and 2, or placebo (*n* = 2) (17). In both the CVD 1204 and CVD 1208 studies, serum samples were obtained before as well as 28 days after vaccination. Only volunteers who consented to the future use of specimens and from whom there were sufficient pre- and postvaccination sera were included in the serological analyses. The clinical studies and laboratory testing were approved by the University of Maryland Institutional Review Board.

### Bacterial growth conditions.

S. flexneri 2a 2457T was streaked on tryptone soy agar (TSA) and incubated overnight at 37°C. Single colonies were propagated in APF Lennox broth (Sigma, St. Louis, MO) to early-log-phase growth (optical density at 600 nm [OD_600_], 0.20 ± 0.02). Bacteria were diluted in phosphate-buffered saline (PBS) for SBA assays or in opsonophagocytic buffer (OPB) consisting of Hanks balanced salt solution supplemented with Ca^2+^ and Mg^2+^ (Gibco, Grand Island, NY) containing 1% gelatin (Sigma) and 5% heat-inactivated fetal bovine serum (Gibco) for OPKA assays.

### SBA assay.

We adapted and optimized an SBA assay based on formats described in the literature (22, 37). Heat-inactivated serum samples were added to 96-well U-bottom plates (Fisher Scientific, Hampton, NH) and serially diluted 2-fold in PBS. S. flexneri 2a 2457T (500 CFU in 10 μl) and 25 μl of baby rabbit complement (BRC; Pel Biologicals, Rogers, AR) were added to the wells (for a total reaction volume of 110 μl and an initial serum dilution of 1:200), and plates were incubated for 1 h at 37°C in a shaker at 200 rpm. The numbers of viable CFU were determined by plating 10 μl of the reaction mixture on TSA and counting the colonies after overnight 37°C incubation. Negative-control wells containing only bacteria and BRC (no serum), as well as a positive-control serum with high bactericidal activity (mean SBA titer ± SD = 2,815 ± 237), were included in each assay. The percentage of killed organisms (per well) was determined by the equation [1 − (number of surviving bacteria/total number of bacteria)] × 100. SBA titers were determined as the reciprocal of the serum dilution that produced 50% bacterial killing using Reed-Muench regression analysis (38). Serum samples were tested and plated in duplicate (for a total of four independent CFU counts); these four data points per sample dilution were averaged for titer calculation.

### OPKA assay.

The OPKA assay was also adapted and optimized using previously described formats (34, 39). Heat-inactivated serum samples were diluted 1:10 in OPB, and 20 μl of this serum dilution was added to 96-well U-bottom plates (Fisher Scientific) and further diluted 2-fold in 10 μl of OPB (for a total of 8 dilutions). S. flexneri 2a 2457T (500 CFU in 10 μl) was added to each well and the serum-bacterium mixture was incubated for 15 min at 37°C. Subsequently, BRC (10 μl) and 1 × 10^5^ dimethylformamide (DMF)-differentiated HL-60 cells (ATCC CCL-240) resuspended in 70 μl were added to the reaction mixture (for a 100-μl total volume and an initial 1:100 serum dilution) and the plates were incubated for 45 min at 37°C, 5% CO_2_. The numbers of viable CFU were determined by plating 10 μl of the reaction mixture on TSA and counting the colonies following overnight incubation at 37°C. Negative-control wells containing bacteria (no serum), BRC, and DMF-differentiated HL-60 cells or bacteria and BRC were included in each assay. A positive-control serum with high OPKA activity (mean titer ± SD = 1,650 ± 124) was also included in each assay. The percentage of bacteria that were phagocytosed and killed per well was determined by the equation [1 − (number of surviving bacteria/total number of bacteria)] × 100. OPKA titers were determined as the reciprocal of the serum dilution that produced 50% bacterial killing based on Reed-Muench regression analysis (38). Serum samples were tested and plated in duplicate; these four data points per serum dilution were averaged for titer calculations.

### ELISA antibody measurements.

Serum IgGs specific for S. flexneri 2a LPS, IpaB, IpaC, and IpaD were measured as previously described (16, 17). To determine VirG-specific IgG, ELISA plates were coated with VirG at 2 μg/ml in carbonate buffer, pH 9.6. Titers were calculated as the inverse serum dilution that resulted in an absorbance value at 450 nm of 0.2 above background and were reported as the number of endotoxin units ml^−1^. Positive and negative controls were included in each assay.

### Statistical analysis.

For both SBA and OPKA, a positive response postvaccination or postchallenge was defined as a 2-fold increase over baseline titers. For IpaB- and VirG-specific IgG titers, a positive response postchallenge was defined as a 4-fold increase over prechallenge titers. Associations among immune responses, measures of disease severity, and vaccination status were evaluated using the Spearman rank correlation coefficient and the Mann-Whitney U test. Logistic regression analysis was performed for disease severity in two categories (DI of 0 or 1 versus DI of 2 or 3), with log-transformed SBA, OPKA, IpaB, and VirG values considered covariates (log-transformed values were used to reduce the influence of extreme data points). A *P* value of ≤0.05 was considered to indicate statistical significance. Analyses were performed using GraphPad Prism 6 (GraphPad Software, Inc., La Jolla, CA) and NCSS 8 and 10 (Number Cruncher Statistical Systems, Kaysville, Utah).

## Supplementary Material

Supplemental material
