# Supplementary material for: Functional and Antigen-Specific Serum Antibody Levels as Correlates of Protection against Shigellosis in a Controlled Human Challenge Study
Source: Clin Vaccine Immunol. 2017 Feb 6;24(2):e00412-16. doi: 10.1128/CVI.00412-16 (PMC5299116; doi:10.1128/CVI.00412-16)
Supplement: Supplemental material [file supp_24_2_e00412-16__index.html]

Supplemental material 

# Functional and Antigen-Specific Serum Antibody Levels as Correlates of Protection against Shigellosis in a Controlled Human Challenge Study

## Supplemental material

- Supplemental file 1 -

  Table S1. Summary of Spearman’s rank correlations between serum antibody titers and disease parameters. Fig. S1. LPS-specific IgG titers do not correlate with disease severity.

  PDF, 124K
