# Supplementary material for: Functional and Antigen-Specific Serum Antibody Levels as Correlates of Protection against Shigellosis in a Controlled Human Challenge Study
Source: Clin Vaccine Immunol. 2017 Feb 6;24(2):e00412-16. doi: 10.1128/CVI.00412-16 (PMC5299116; doi:10.1128/CVI.00412-16)
Supplement: Supplemental material [file CVI.00412-16_zcd999095443s1.pdf]

Supplementary Table 1. Summary of Spearman's rank correlations between serum antibody titers and disease parameters.

	Pre-challenge antibody titers correlation coefficient (r) and p-values							
	**SBA**		**OPKA**		**IpaB**		**VirG**	
	r	p	r	p	r	p	r	p
**dysenteric stools**	-0.54	0.0016	-0.52	0.0005	-0.58	<0.0001	-0.43	0.01
**loose stools**	-0.46	0.01	-0.47	0.01	-0.62	0.0002	-0.44	0.02
**stool volume (L)**	-0.41	0.03	-0.39	0.04	-0.60	0.0004	-0.49	0.0044
**maximum temp (^{0}F)**	-0.36	0.06	-0.32	0.09	-0.70	0.0006	-0.64	0.0001

Supplementary Figure 1.

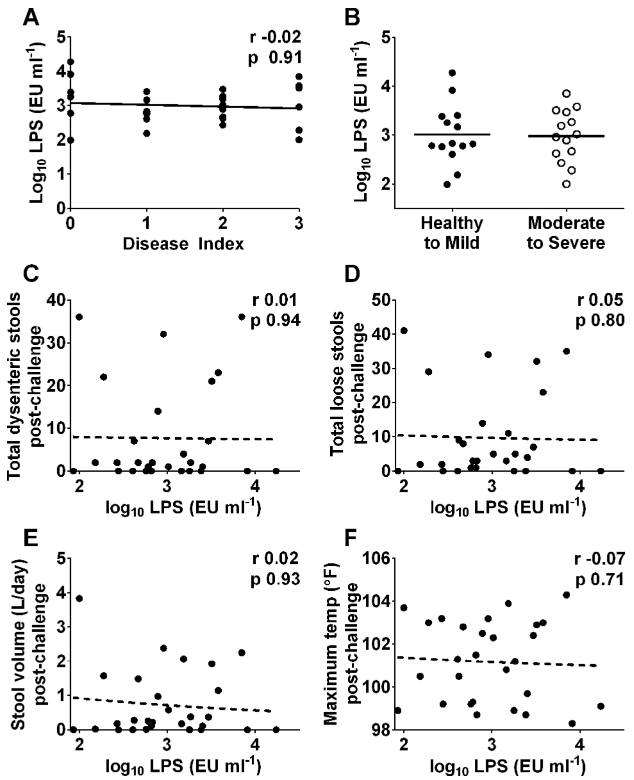

LPS-specific IgG titers do not correlate with disease severity. LPS-specific IgG titers were measured in serum from EcSf2a-2 vaccinated (n=15) and naïve (n=13) volunteers before challenge with wild-type *S. flexneri* 2a. LPS-specific IgG titers (A) were compared with DI post-challenge using Spearman-rank correlation (r- and p-value indicated on plot). Titers were further grouped and compared based on disease severity (healthy to mild and moderate to severe) using Mann-Whitney U-test, $p>0.05$ (B). LPS-specific IgG titers were then compared with disease parameters post-challenge: dysenteric stools (C), loose stools (D), stool volume (E) and body temperature (F) using Spearman-rank correlation (r-value coefficient).
